# Parameters of stochastic models for electroencephalogram data as biomarkers for child’s neurodevelopment after cerebral malaria

**DOI:** 10.1186/s40488-018-0086-7

**Published:** 2018-12-29

**Authors:** Maria A. Veretennikova, Alla Sikorskii, Michael J. Boivin

**Affiliations:** 1 Department of Statistics and Data Analysis, Faculty of Economic Science, National Research University, Higher School of Economics, Shabolovka 28/11, 9, Moscow, 19049 Russia; 2 Department of Psychiatry and Department of Statistics and Probability, Michigan State University, 909 Wilson Road, East Lansing, 48824 MI USA; 3 Department of Psychiatry and Department of Neurology and Ophtalmology, Michigan State University, 909 Wilson Road, East Lansing, 48824 MI USA

**Keywords:** 60E05, 62M10, 62P10, Student processes coma EEG wavelets regression regularization

## Abstract

The objective of this study was to test statistical features from the electroencephalogram (EEG) recordings as predictors of neurodevelopment and cognition of Ugandan children after coma due to cerebral malaria. The increments of the frequency bands of EEG time series were modeled as Student processes; the parameters of these Student processes were estimated and used along with clinical and demographic data in a machine-learning algorithm for the prediction of children’s neurodevelopmental and cognitive scores 6 months after cerebral malaria illness. The key innovation of this work is in the identification of stochastic EEG features that can serve as language-independent markers of the impact of cerebral malaria on the developing brain. The results can enhance prognostic determination of which children are in most need of rehabilitative interventions, which is especially important in resource-constrained settings such as sub-Saharan Africa.

## Introduction

Cerebral malaria (CM) affects over half a million people annually and has high prevalence in sub-Saharan Africa. Different sources indicate distinct mortality rates, but for children it is above 40 percent (Idro et al. 2010). For those who survive, the sequelae could include neurodevelopmental impairments and metabolic disturbances.

During CM the red blood cells are parasitized, most often by *Plasmodium Falciparum*. *P. Vivax* and *P. Knowlesi* are also known to cause severe malaria, but unlike *P. Falciparum* they do not lead to coma (WHO 2014). Coma is the principal diagnostic difference between severe malaria and CM, with lumbar puncture ruling out bacterial central nervous system (CNS) infection or other cause of coma beyond severe malaria. CM is specific to *P. Falciparum* and is distinctive from other forms of malaria because of the sequestration of infected red blood cells in the microvasculature of the brain and compromise of the blood/brain barrier contributing to an immunopathogenic inflammatory cascade. This cascade together with ischemic and metabolic effects cause coma and often seizures during illness and contribute to the neuropathogenic basis of subsequent neurological and neurocognitive sequelae (John et al. 2008). Since coma is defined as prolonged unconsciousness and unresponsiveness (usually between 1 h and 4 weeks), clinical language-independent data provide the only opportunity to gauge the extent to which brain injury may impact subsequent neurodevelopment and cognitive function.

Today there is a number of different techniques for the statistical analysis of the brain. Despite some limitations, EEG is still widely used as a noninvasive way to monitor patients, predict seizure onsets and to determine the amount of activity in near-death states. In Awal et al. (2016) the authors use EEG features to predict neurodevelopmental outcomes for term infants with hypoxic ischaemic encephalopathy (HIE). The association between brain activity during coma and trauma outcomes was investigated in Malagurski et al. (2017) and Juan et al. (2015). Statistical analysis of EEG has been used to identify quasi-brain-death from coma (Li et al. 2014) and to confirm brain death (Chen et al. 2008).

Much of the work on the analysis of EEG data has focused on classification (Kirch et al. 2015; Piryatinska et al. 2009; Temko et al. 2011) or prediction of seizures (Duncan et al. 2013) without specification of the model for the underlying stochastic process. The use of spectral methods for the EEG time series, while popular, is problematic due to the evidence of non-stationarity of the process (Ignaccolo et al. 2009). In this paper we propose a stochastic model for the EEG time series, where for each frequency band the increment process is assumed to be a Student process, realized as Lévy driven Ornstein-Uhlenbeck-type process. The parameters of the Student marginal distribution are estimated and entered into a machine learning algorithm to test their association with children’s neurodevelopmental and cognitive scores 6 months after cerebral malaria illness. The use of the Student distribution parameters markedly improves explained variation of neurodevelopment and cognition compared to using only demographic and clinical characteristics including plasma and cerebrospinal fluid biomarkers, or prediction based upon frequently used traditional EEG features. Identification of biomarkers such as the parameters of stochastic models for the EEG data has the potential to enhance diagnostic and prognostic determination by complementing the very limited clinical expertise in neurologists able to read and interpret EEG in resource constrained settings. Further, language-independent markers of neurodevelopment and cognition based on stochastic features of EEG data can complement the limited expertise available in sub-Saharan Africa for developmental and cognitive evaluations.

## Dataset and preprocessing

Data used in this analysis were collected during the observational study of the pathogenesis of severe malaria (cerebral malaria (CM) and severe malarial anemia (SMA)) in surviving children, along community control children from their households who did not have a history of severe malaria (Bangirana et al. 2016). The study was performed at Mulago National Referral and Teaching Hospital in Kampala, Uganda in 2008−2015. Children with cerebral malaria, severe malarial anemia, or community control children were enrolled if they were between 18 months and 12 years of age. Cerebral malaria was defined as: 1) coma (Blantyre Coma Score [BCS] ≤2; 2) Plasmodium falciparum on blood smear; and 3) no other known cause of coma (e.g., hypoglycemia-associated coma reversed by glucose infusion, meningitis, or a prolonged postictal state). Children were enrolled after obtaining written informed consent in the local language from their parent(s) and signed assent from children 7 years of age and older.

The observational study was approved by the Institutional Review Boards of the Makerere University School of Medicine and the University of Minnesota. Data from CM malaria children were included in this study as EEGs were done only for them and not SMA or community control children. Data from community controls were used to create the z-scores of neurodevelopmental and cognitive outcomes of the CM children as described below.

### 2.1 EEG data

MATLAB software was used for the processing of the EEG data set that comprised the standard 10–20 EEG recordings for 78 children with the sampling rate of 500 Hz and the average record duration of 30 min. Persyst software (Persyst, Prescott, USA) was used to remove artifacts due to breath, muscle movement and heartbeat from the raw EEG data. For most children there were 19 channels, which means the electrodes were not located very densely, so there was no necessity to use the average reference. Hence we’ve chosen CZ to be the reference electrode to avoid the laterality bias. CZ is one of the predominant choices for a reference (Teplan 2002). Then we used EEGLAB (Makeig and Delorme 2004) to identify problematic channels based on properties of their voltage measurements, leaving 16 channels for analysis and excluding three (PZ-CZ, C4-CZ, O2-CZ). Data for several of the included 16 channels had substantial numbers of zero observations, which could be due to poor connection between the electrode and the skin. For these channels we extracted features described below with and without zeros, with the rationale that if a feature resulted from an artifact and was not important, it would not be selected by the machine learning algorithm.

We used Daubechies wavelets for splitting the clean signal into frequency bands. Daubechies orthogonal wavelets have a number of vanishing moments, which is used as an index for referencing, e.g. the standard notation Db4 means Daubechies wavelet with 4 vanishing moments. Research indicates particular suitability of Db4 for statistical analysis of EEG. We examined the relative average mean squared error (MSE) between the wavelet signal approximation and the actual signal for different Daubechies wavelets. For these data Db4 yielded the reconstruction error or order 10^−9^, which is sufficiently low. Also, Db4 frequency band separation resulted in frequency intervals which are very close to the traditional frequency ranges: delta, theta, beta, alpha and gamma bands (Daubechies 1992), see Table 1.

**Table 1:** Frequency band correspondence

Traditional	Db4 band’s central frequency
Delta 0−3.5 Hz	2.7 Hz
Theta 3.5−7.5 Hz	5.57 Hz
Alpha 7.5−13 Hz	11 Hz
Beta 13−30 Hz	22.3 Hz
Gamma >30 Hz	four subsequent bands

Due to occasional spikes and irregular patterns in the original time series, the idea was not to split them into epochs as it is often done (Fraschini et al. 2016). We hypothesized that some of the statistical features, such as the frequency of flat line measurements relative to the whole EEG record, could be useful as explanatory variables for neurodevelopment and cognition, whilst dividing the record into epochs would complicate extraction of useful information.

Empirical studies show that a low activity level in the gamma frequencies is closely related to the coma state (Chen et al. 2008), and generally, gamma band oscillations are thought to be related to higher cortical functioning, such as consciousness, memory, perception and learning (Uhlhaas et al. 2009). In Deng et al. (2015) it was shown that EEG gamma band activity characteristics are associated with the outcomes of targeted temperature management for brain recovery after cardiac arrest. On the other hand, it is also advised to exclude the highest EEG gamma frequency bands from the analysis, because it is most likely to be noise, rather than the real deterministic signal. In view of these recommendations and the goal of this research to investigate stochastic features of EEG, we excluded only the highest gamma band (D1), keeping the rest. Our rationale was that if the other gamma bands (D2, D3, and D4 in Daubechies’ frequency band notation) were indeed useless for the prediction of post-comatose neurodevelopmental and cognitive scores, then this would be empirically determined in statistical algorithms for the extraction of important features.

### 2.2 Measures of neurodevelopment and cognition

Children had neurodevelopmental assessments (appropriate for those 5 years of age or younger) or cognitive assessments (appropriate for children over 5 years of age) a week after discharge from the hospital (or at enrollment for community control children) and then at 6 and 12 months after enrollment. Data from the assessment at 6 month post-enrollment were used for this analysis.

**Neurodevelopmental assessment for children 5 years of age or younger.** The Mullen Scales of Early Learning (MSEL) (Mullen 1995) were used to quantify neurodevelopment. MSEL is based on a comprehensive test assessing specific developmental domains: visual reception, gross motor skills, fine motor skills, receptive language, and expressive language. A composite score derived from standardized t-scores of the four domains (excluding gross motor) provides a measure of *g*, the general measure of fluid intelligence.

**Cognitive assessment for children over 5 years of age.** The Kaufman Assessment Battery for Children, second edition (KABC-II) (Kaufman and Kaufman 2004) evaluates sequential and simultaneous processing, learning, reasoning, and crystallized intellectual ability (knowledge). The knowledge subscale was not administered because it was not suitable in this setting (Bangirana et al. 2009). Summation of scores for the domains of sequential processing, simultaneous processing, learning, and planning yielded the Mental Processing Index (MPI) which was the measure of overall cognitive ability in this age group.

The United States of America (USA) norms were used to arrive at the MSEL composite *g* score and the KABC-II MPI score, because using such norms to adjust for the child’s age was necessary to compute these global measures. To obtain a single measure of neurodevelopment or cognition for all children regardless of age, we computed the means and standard deviations of the age appropriate measures, the MSEL composite or the KABC-II MPI, among the community control children. Then for CM children the z-scores in each age group were computed by subtracting the means and dividing by the standard deviation of the community control children.

### 2.3 Other measures

Home Observation for the Measurement of the Environment (HOME) (Bradley and Caldwell 1979) is a composite measure designed to assess the quality and quantity of stimulation that the child is exposed to in their home environment. A total HOME score was generated by summing the number of *“yes”* responses to a checklist of items; higher HOME scores indicate higher quality of home environment.

Demographic and anthropometric data included age, sex, height-for-age and weight-for-age z-scores computed using the World Health Organization reference norms (Wold Health Organization Growth Standards 2009). Socioeconomic status (SES) was assessed using a checklist of material possessions, housing quality, cooking resources and water accessibility. Clinical variables and biomarker panels from plasma and cerebrospinal fluid were collected during each child’s hospitalization for CM.

## Creating the feature matrices

### 3.1 Non-EEG features

The non-EEG features included demographic and anthropometric data, SES and HOME scores, the Blantyre Coma Score, and plasma and cerebrospinal fluid biomarker panels, for a total of 54 potential explanatory variables.

### 3.2 Commonly used EEG features

We have evaluated 362 EEG features that have been commonly used in the past analyses of EEG data. Presence of seizures was reflected by binary variable that was defined using Persyst software indicators (Persyst, Prescott, USA). Frequencies of peaks in the original cleaned time series differing from the nearest measurements from both sides by 1/3, 1, 2 and 3 standard deviations were calculated and denoted by fp1/3, fp1, fp2 and fp3, respectively. Proportion of flat line EEG for each of the 16 channels was evaluated for the original cleaned time series.

For delta (a7), theta (D7), alpha (D6), beta (D5) and gamma (D4, D3, D2) frequency bands for each channel, we calculated amplitude variances and Shannon time entropy using wavelets (De Oliveira 2015; Smolentsev 2014). This version of entropy is defined in MATLAB as

$$\begin{array}{@{}rcl@{}} S\left(x_{i}\right)=-\sum\limits_{i=1}^{N-1}x_{i}^{2}\log \left(x_{i}^{2}\right) \end{array} $$

where *x*_*i*_ is the *i*-th measurement in the time series for the signal (MATLAB wavelet packet). Relative frequency band energy was defined as the sum of wavelet coefficients divided by the total sum of the coefficients for all the frequency bands (Smolentsev 2014, Chapter 3.10).

Hjorth complexity and mobility parameters (Hjorth 1970) were calculated for the entire time series based on the second moment as well as the first and second order differences.

### 3.3 New stochastic features

**Fig. 1:**
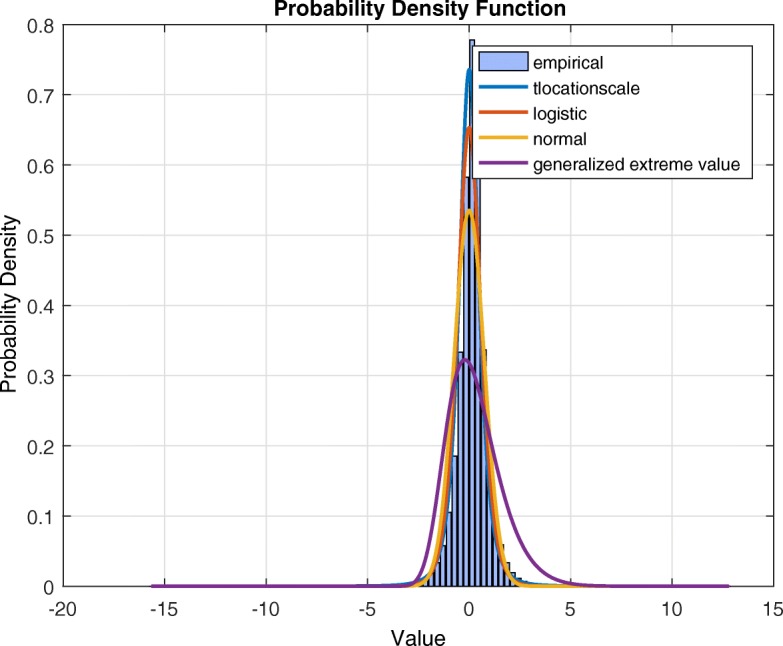
Alpha frequency band

After splitting the EEG time series into frequency bands using Daubechies wavelets, for each frequency band we constructed the histograms for the increment process at different time blocks, and evaluated sample means and variances. The histograms were consistently approximately bell-shaped but with peaks higher and tails heavier than normal (Fig. 1).

The means were consistently close to zero, and the variances ratios for different time blocks fell within a rule of thumb range of [0.25, 4] (Montgomery 2012). Based on these empirical features, we selected a modeling approach that uses a stationary stochastic process for the increments of time series. To reflect the leptokurtic distribution seen in the data, we propose a stationary Student process as a model for the increments of the EEG time series for each frequency band. The symmetric scaled Student marginal distribution has the density

1 $$\begin{array}{@{}rcl@{}}  f_{\nu,\delta}(x)=\frac{\Gamma\left(\frac{\nu+1}{2}\right)}{\delta\sqrt{\pi}\Gamma\left(\frac{\nu}{2}\right)}\left(1+\left(\frac{x-\mu}{\delta}\right)^{2}\right)^{-\frac{\nu+1}{2}}, \ x\in \mathbb{R}, \end{array} $$

*ν*>0 is degrees of freedom, *δ*>0 is a scale parameter, $\mu \in \mathbb {R}$ is location parameter. We denote this distribution by *T*_*μ*,*δ*,*ν*_. The expectation exists when *ν*>1, the variance exists when *ν*>2, and generally the *n*-th central moment exists when *ν*>*n*.

There are several processes with the Student marginal distribution (Heyde and Leonenko 2005), of which we chose the Lèvy driven Ornstein-Uhlenbeck (OU)-type process as the process with the least restrictive potential parameter range. We have considered Student diffusion process (Leonenko and Suvak 2010) as another possible model. However, the mean reversion term needs to be included in the stochastic differential equation (SDE) defining a stationary Student diffusion process. Thus the mean has to exist, restricting the parameter range to *ν*>1. The parameter estimates based on these data did not support such restriction across all channels. The Student OU type process provides a flexible model for the increments of the EEG data, and is the solution of SDE

2 $$\begin{array}{@{}rcl@{}}  dX(t) = -\lambda X(t)dt +dY(\lambda t), \quad t\ge 0 \end{array} $$

where {*Y*(*t*), *t*≥0} is the background driving Lévy process (BDLP). This process is defined by specifying the distribution of *Y*(1) through the cumulant transform

3 $$\begin{array}{@{}rcl@{}} \log e^{ i\zeta Y(1)}=i\zeta \mu -\delta |\zeta |\frac{K_{\nu/2-1}(\delta |\zeta|)}{{K_{\nu/2}(\delta |\zeta|)}}, \quad \zeta \in \mathbb{R}, \,\, \zeta \ne 0, \end{array} $$

where *K*_*s*_ is the modified Bessel function of the third kind:

$$\begin{array}{@{}rcl@{}} K_{s }(x)=\frac{1}{2} \int_{0}^{\infty} u^{s -1}\exp{ \left[\frac{1}{2}x\left(u+\frac {1}{u}\right)\right]}du,\,\, x>0,\,\,s \in \mathbb{R}. \end{array} $$

Since the Student distribution is self-decomposable, the distribution of *Y*(1) is infinitely divisible, and the Student OU-type process exists, as formally stated in the following Theorem (Heyde and Leonenko 2005).

#### **Theorem 1**

There exists a strictly stationary stochastic process {*X*(*t*), *t*≥0} that has the marginal *T*_*μ*,*δ*,*ν*_ distribution with the density function (1). The process solves SDE (2) for any *λ*>0. The BLDP {*Y*(*t*), *t*≥0} has the cumulant transform (3). The solution is given by

$$\begin{array}{@{}rcl@{}} X_{t}=e^{-\lambda t} X_{0} +e^{-\lambda t} \int_{0}^{t} e^{\lambda s} dY(\lambda s). \end{array} $$

Note that parametrization in (2) is such that the marginal distribution of the Student OU-type process *X*(*t*) does not depend on *λ*. If *ν*>1, then the first moment of the marginal distribution exits, and $ \mathbb {E} X(t)=\mu $. If *ν*>2, then the correlation function exists and depends only on the parameter *λ*, namely for 0<*s*<*t*

$$\begin{array}{@{}rcl@{}} \text{corr} (X(s), X(t))=e^{-\lambda (t-s)}. \end{array} $$

In this work we used the first-order properties of the process *X*(*t*) reflected by the parameters of the marginal distribution. We set *μ*=0 based on the empirical evidence as all histograms were centered on zero with virtually no variability in this respect among EEG channels. We used quasi-likelihood estimation (Heyde 1997) to evaluate the parameters *ν* and *δ* of the stationary Student process. It was possible to use this approach because of the specification of the distribution. A general method for estimation of the tail parameter (*ν* in this case) without specifying the marginal distribution is discussed in Grahovac et al. (2015) and could be used in other applications. Quasi-likelihood estimation was performed for the lowest four frequency bands for 15 channels, resulting in 120 features. Additional 30 features were derived by repeating the parameter estimation for 15 channels delta frequency band containing excess zeros potentially due to the disconnection of the electrode from the skin. Note that in this set of features there is no CZ-CZ channel at all. The estimated 150 parameters were used as features in the matrix of potential explanatory variables for the neurodevelopment and cognition 6 months post CM illness. We refer to these features as stochastic features since they were derived based on the proposed stochastic process model.

## Data analysis

Three feature matrices X with 78 rows each, one containing 54 non-EEG features (columns), the second containing 362 traditional EEG features, and the third containing 150 new stochastic EEG features, were prepared as described above for the entry into a machine learning algorithm to predict the neurodevelopmental and cognitive scores of children surviving cerebral malaria. Out of 54 columns for non-EEG features 20 had at least 19 missing values. The maximum number of empty entries in a column of the non-EEG part of the feature matrix was 23. The pattern of missing data could not be assumed to be missing at random (MAR), because some of the data were missing due to clinical reasons. Therefore we used imputation method that is not dependent upon the MAR assumption. Soft Impute is a matrix completion method based on Singular Value Decomposition (SVD) of a matrix (Mazumder et al. 2010). Application of this algorithm requires the following assumption to hold: the rank of the approximating matrix *rank*(*Z*)<<*min*(*n*,*p*), which is reasonable in our case. This assumption makes sense due to the nature of features, which may be grouped by correlation into a smaller number of clusters due to inherent synchrony between channels. We present the central lemma behind this method for completeness.

### **Lemma 1**

Suppose the matrix *W*_*m*×*n*_ has rank *r*. The solution to the optimization problem

4 $$\begin{array}{@{}rcl@{}} \min_{Z} \left(\frac{1}{2}\|W-Z\|^{2}_{F} + \lambda\|Z\|_{*}\right) \end{array} $$

is given by $\hat {Z}=S_{\lambda }(W)$, where *S*_*λ*_(*W*)=*UD*_*λ*_*V*^*T*^, with *D*_*λ*_=diag[(*d*_1_−*λ*,⋯,(*d*_*r*_−*λ*)_+_], where *UDV*^*T*^ is the SVD of W, *D*=diag[*d*_1_,⋯,*d*_*r*_], and *t*_+_= max(*t*,0).

Here ∥*A*∥_*F*_ is the Frobenius norm of a matrix A, whilst ∥*A*∥_∗_ is the sum of the singular values of the matrix A. In our case, in computing the Frobenius norm in (4) we only look at the pairs of indices (*i*,*j*), for which there are no missing values. The algorithm implemented in SoftImpute iteratively updates the matrix *Z* through the use of this lemma, until convergence is reached in approximating the matrix of interest. R was used for the imputation of missing values.

Machine learning was performed using the Elastic Net technique that solves the following optimization problem:

5 $$\begin{array}{@{}rcl@{}} \min_{\bar{\beta}} \left(\sum\limits_{i=1}^{n}\left(y_{i} - \sum\limits_{j=1}^{p}\beta_{j}x_{ij}\right)^{2} + \alpha \times l_{1} \sum\limits_{j=1}^{p}|\beta_{j}| + 0.5 \times \alpha \times (1- l_{1}) \sum\limits_{j=1}^{p}\beta_{j}^{2} \right), \end{array} $$

Elastic Net was chosen because it is more suitable than the Least Absolute Shrinkage and Selection Operator (LASSO) in face of multicollinearity (Oyeyemi et al. 2015), which we expected among the selected features. There are two hyper-parameters, and leave-one-out cross validation (LOOCV) was used to select the optimal pair. We have also used LOOCV for the estimation of the mean squared error in the prediction models produced by the algorithm. The Elastic Net algorithm was implemented using Python 3 with Anaconda.

The non-parametric missForest technique for matrix completion (Stekhoven and Bühlmann 2012) is based on averaging over a random forest of regression trees and was used for additional validation of the results. Trees were built based on observed and bootstrapped parts of the training data set.

## Results

### 5.1 Imputations

We have run over a grid of different parameter values for *α*, considering powers of 10, starting with *α*=0.00001 and finishing with *α*=1000, whilst for the *l*_1_ ratio coefficient we considered 7 possible values starting with 0.0001 and finishing with 1. In all models, setting the regularization parameter *λ*=100 in SoftImpute gave better mean squared error (MSE) than other values of *λ* and resulted in the least complex model, after applying the Elastic Net following the matrix completion. This value is used in reporting of the results.

### 5.2 Results from elastic net for three sets of features

Table 2 summarizes the results of applying the regression methods listed in the previous section.

**Table 2:** Elastic Net - best results, after Soft Impute with *λ*=100

Feature set	LOOCV MSE	Number of nonzero coefficients	Sample features with non-zero coefficients
54 anthropomentic, socio-economic, and medical non-EEG features	0.3982	15	Weight, hemoglobin level, weight, BCS, the HOME score, white blood cell count, cerebrospinal fluid levels of interleukin (IL)-1 receptor antagonist (RA), IL-6, RANTES (an acronym for Regulated on Activation, Normal T Expressed and Secreted), IL-8, and plasma levels of vascular endothelial growth factor and von Willebrand factor.
362 traditional EEG features in frequency bands	0.5285	62	Features include: fp2 in T6-CZ, FZ-CZ and P4-CZ, fp1 F4-CZ, wave energy for theta in channel T5-CZ, variance in theta for FP2-CZ, variance in alpha frequency band for T5-CZ.
150 stochastic features for increment processes in frequency bands	0.1511	85	12 coefficients have the absolute values over 0.5. Top 5 coefficients are for the channels FP2-CZ, O1-CZ, T6-CZ, FZ-CZ. Channels F8-CZ and T6-CZ appear most often among the 12 top coefficients by absolute value, 4 and 3 times respectively. Four of the top 12 are for fitting a stochastic process model for the a7 frequency band.

The lowest LOOCV MSE of 0.15 was obtained with *α*=0.001, *l*_1_ ratio =0.5 in the objective function subject to minimization:

6 $$\begin{array}{@{}rcl@{}} \frac{1}{2*78}\|y-Xw\|_{2}^{2}+ \alpha*\text{l1 ratio}*\|w\|_{1}+0.5*\alpha*(1-\text{l1 ratio})*\|w\|_{2}^{2} \end{array} $$

with leave-one-out cross-validation. This combination of *α* and *l*_1_ ratio was the best across combinations described in the Section 4.

Similarly to the result for the matrix with 150 features after SoftImpute, using the random forest technique for matrix completion and again, those features in the top 5 in feature importance by Breiman were for the channels FP2-CZ, O1-CZ, F7-CZ, T6-CZ, whilst F8-CZ and T6-CZ appeared most often in the top 12 non-zero coefficients by absolute value. So this result almost copies the outcome with the Elastic Net after SoftImpute with *λ*=100 and yields a marginally different Elastic Net LOOCV MSE value. This was anticipated and confirms validity of the matrix completion method for such data.

## Conclusions

We conclude that stochastic modeling brings a noticeable improvement in explaining the variation in neurodevelpmental and cognitive outcomes of children 6 months after surviving cerebral malaria. Stochastic features alone do an even better job than the tested sets of medical non-EEG or traditional EEG features which are not based on stochastic models for the underlying time series.

Regarding medical non-EEG biomarkers, only tumor necrosis factor alpha (TNF-alpha) in cerebrospinal fluid but not in plasma was predictive of 6-month later cognitive scores of children older than 5 years, but not of neurodevelopment of younger children (Shabani et al. 2017). So our finding of TNF-alpha not being among top predictors is in line with the developing literature on the role of biomarkers collected at the time of acute illness in predicting later neurodevelopment and cognition in children. This paper extends the results of Shabani et al. (2017) to testing more than one biomarker using modern statistical and probabilistic methods. We have identified biomarkers that can be further considered in future research as potentially important prognostic factors for neurodevelopment and cognition.

Regarding the traditional EEG features, their performance in explaining the variation in neurodevelopmental and cognitive outcomes was inferior to that of anthropometric, socio-economic, and non-EEG medical features. This finding may be due to the fact that these features are not based on underlying stochastic models. For example, the traditional computation of Shannon’s entropy build into software assumes that the underlying stochastic process is stationary, which could be reasonable in some populations (Piryatinska et al. 2009), but is at odds with other literature (Ignaccolo et al. 2009). Whether or not the underlying stochastic process is stationary in a given population is an empirical question that needs to be addressed in methodology of analyzing EEG data. For the population of Ugandan children in coma from cerebral malaria, we have found that the assumption of stationarity of the time series was unreasonable, while for the increment process it was. Further, stochastic modeling for the increment process had clear advantages, as seen from our results.

When considering the stochastic features for the increment process, the combination of channels for which the stochastic features proved to be particularly useful is FP2-CZ, 01-CZ, T6-CZ, FZ-CZ. Parameters from channels located on the right side dominated the most important features. Taken as a group, these locations are associated with visual and attention processes that are related to visual-spatial simultaneous processing working memory. It would be interesting to see if these channels also arise as important in relation to child neurodevelopment and cognition in other infectious diseases that could affect the brain.

## Abbreviations

BCS: Blantyre coma score
CM: Cerebral malaria
Dbi: Daubechies wavelet with *i* vanishing moments
D1, …, D7, a7: Frequency bands from the highest gamma (D1) to delta (a7), see the table
EEG: Electroencephalogram
fp1/3, fp1, fp2 and fp3: Frequencies of peaks in the original cleaned time series differing from the nearest measurements from both sides by 1/3, 1, 2 and 3 standard deviations were calculated
HOME: Home observation for the measurement of the environment
Hz: Hertz
KABC-II: Kaufman assessment battery for children, second edition
LOOCV MSE: Leave-one-out cross-validation mean squared error
MAR: Missing at random
MPI: Mental processing index
MSEL: Mullen scales of early learning
SDE: Stochastic differential equation
SES: Socioeconomic status
SMA: Severe malarial anemia
SVD: Singular value decomposition
TNF-alpha: Tumor necrosis factor alpha
